# Proinflammatory Markers, Chemokines, and Enkephalin in Patients Suffering from Dry Eye Disease

**DOI:** 10.3390/ijms19041221

**Published:** 2018-04-17

**Authors:** Pierre Nicolle, Hong Liang, Elodie Reboussin, Ghislaine Rabut, Elise Warcoin, Françoise Brignole-Baudouin, Stéphane Melik-Parsadaniantz, Christophe Baudouin, Antoine Labbe, Annabelle Reaux-Le Goazigo

**Affiliations:** 1 Department of Ophthalmology III, Quinze-Vingts National Ophthalmology Hospital, F-75012 Paris, France; pierrenicolle86@hotmail.com (P.N.); lianghongfr@yahoo.fr (H.L.); ghislaine.rabut@gmail.com (G.R.); cbaudouin@15-20.fr (C.B.); dr.antoinelabbe@gmail.com (A.L.); 2 Quinze-Vingts National Ophthalmology Hospital, DHU Sight Restore, INSERM-DGOS CIC 1423, F-75012 Paris, France; 3 Sorbonne Université, INSERM, CNRS, Institut de la Vision, F-75012 Paris, France; elodie.reboussin@inserm.fr (E.R.); elisewarcoin@gmail.com (E.W.); fbaudouin@15-20.fr (F.B.-B.); Stephane.Melik-Parsadaniantz@inserm.fr (S.M.-P.); 4 Department of Ophthalmology, Ambroise Paré Hospital, APHP, University of Versailles Saint-Quentin en Yvelines, F-92100 Boulogne-Billancourt, France

**Keywords:** dry eye disease, ocular pain, enkephalin, chemokines, inflammation

## Abstract

Dry eye symptoms are among the leading complaints in ophthalmology. Dry eye disease (DED) is associated with significant pain affecting quality of life. Cellular and molecular mechanisms underlying ocular pain associated with DED are not fully understood. In this study, we investigated the ocular surface of patients with DED using in vivo confocal microscopy (IVCM) to quantify corneal nerve density and its relation with corneal inflammation. Gene expression of the proinflammatory markers HLA-DR, IL-6, CXCL12, and CCL2 and the receptors CXCR4 and CCR2, as well as PENK (enkephalin precursor), was therefore quantified in conjunctival impression cytology specimens. Thirty-two patients with DED and 15 age-matched controls were included. Subbasal nerve density was significantly lower in DED patients compared to controls. IVCM analysis revealed that DED patients had a significantly higher corneal dendritic cell density compared to controls. Conjunctival impression cytology analysis revealed that HLA-DR, IL-6, CXCR4, and CCL2/CCR2 mRNA levels were significantly increased in DED patients compared to controls, whereas PENK mRNA levels were significantly decreased. Similar results were obtained in vitro on immortalized human conjunctiva-derived epithelial cells challenged with osmotic stress that mimics the DED condition. These results demonstrate that proinflammatory molecules and endogenous enkephalin have opposite gene regulation during DED.

## 1. Introduction

Symptoms of dry eye disease (DED) include visual disturbance and variable intensity of pain and signs of discomfort that may become permanent and thus significantly affect quality of life [1,2]. It has been previously shown that the impact of moderate to severe DED on quality of life is equivalent to that of stage III/IV angina pectoris [3]. Nevertheless, the pathophysiological mechanisms of ocular pain in dry eye remain largely unknown. Moreover, a decorrelation between symptoms and clinical signs is classically described [4]. This lack of correlation between clinical signs and symptoms emphasizes the complex mechanisms of symptoms in DED and the difficulty of relieving them.

Ocular surface nociceptive messages result from the stimulation of nociceptors located in the nerve terminals of trigeminal neurons innervating the conjunctiva and the cornea. Accumulative data have confirmed that inflammation of the ocular surface plays an important role in DED [5] and inflammatory markers such as human leukocyte antigen (HLA)-DR [6], interleukin 6 (IL-6), and chemokine (C-C motif) ligand 2 (CCL2) [7] have been found in the conjunctival and corneal epithelial cells of patients with dry eye. Recent research from our group has shown that the chemokine CCL2 and the stromal cell-derived factor 1 (SDF1), also known as C-X-C motif chemokine 12 (CXCL12), with their respective receptors (CCR2 and CXCR4), participate in the modulation of nociceptive information [8,9,10]. In addition to their pro-nociceptive actions, these CCL2 and CXCL12 chemokines are also able to blunt peripheral morphine analgesia by desensitizing opioid receptors [11]. This concept is of particular importance because it is well established that peripheral inflammatory pain can be controlled by endogenous opioid encephalin [12] produced by immune cells in contact with nociceptive fibers. Once released by immune cells [13,14], this endogenous opioid activates opioid receptors on sensory neurons to inhibit pain information with an analgesic action [12,13,14]. This mechanism has already been demonstrated during the inflammation of peripheral tissues such as skin [13,15,16], but to our knowledge, the gene expression of this molecule has not yet been investigated during dry eye.

This study evaluated whether dry eye-associated ocular pain is associated with a modulation of the expression of the main endogenous opioid (enkephalin), *CXCL12* and *CCL2* chemokines, and their respective receptors *CXCR4* and *CCR2*, respectively, as well as inflammatory markers (HLA-DR and IL-6) ex vivo in the ocular surface and in vitro in conjunctiva-derived epithelial cells. The purpose of the present study was also to provide solid evidence of an unbalanced gene expression of pro-inflammatory markers and endogenous enkephalin in DED.

## 2. Results

### 2.1. Clinical Analysis

Patients in the DED group had suffered from dry eye for 5.0 ± 4.7 years. The OSDI score was higher in the DED group than in controls (67.8 ± 21.4 vs. 6.2 ± 4.4; *p* < 0.05). According to the visual analog scale, all patients from the DED group had painful symptoms related to dry eye (mean score, 6.1 ± 2.9). The Schirmer test was significantly decreased in the DED group compared to the control group (12.5 ± 8.8 mm vs. 24.7 ± 4.1 mm; *p <* 0.05). TBUT was also significantly decreased in the DED group (5.4 ± 3.3 s vs. 14.4 ± 2.3 s; *p <* 0.05). The Oxford score was significantly higher in the DED group (1.4 ± 1.6 AU vs 0.0 ± 0.0 for controls). The clinical results are summarized in Table 1.

### 2.2. In Vivo Confocal Microscopy Analysis

**Figure 1:**
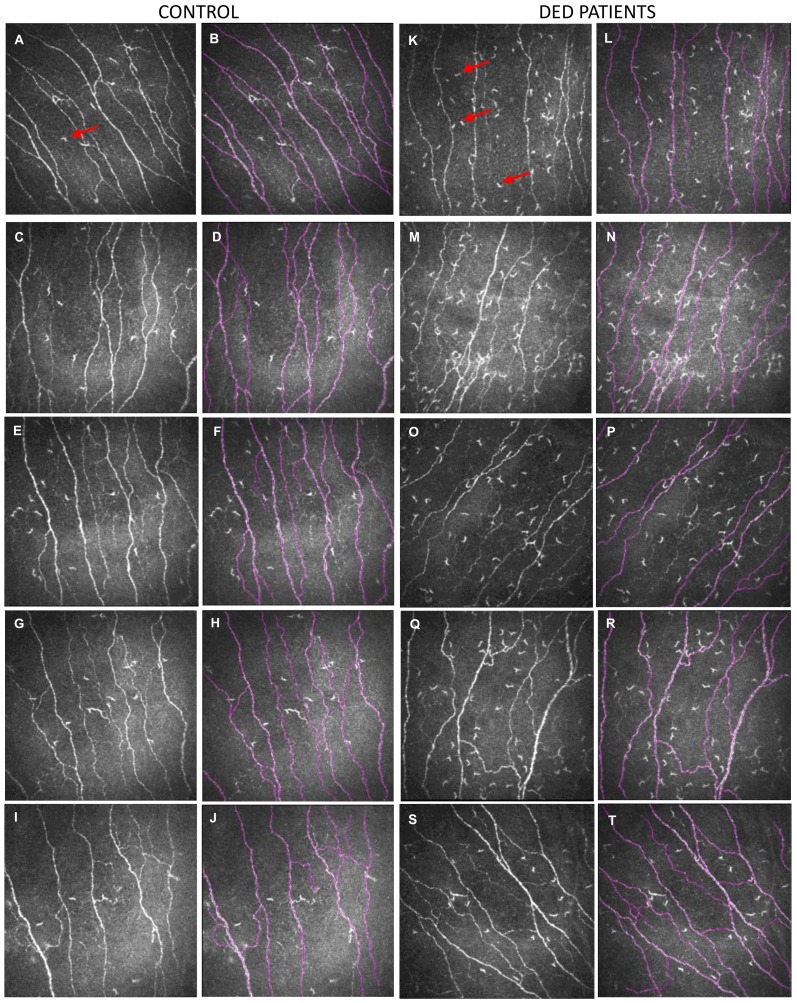
Representative in vivo confocal microscopy (IVCM) images (400 × 400 µm) of corneal subbasal nerves and subbasal dendritic cells from five control subjects (**A**,**C**,**E**,**G**,**I**) and five patients with dry eye disease (DED) (**K**,**M**,**O**,**Q**,**S**). Images (**B**,**D**,**F**,**H**,**J**,**L**,**N**,**P**,**R**,**T**) illustrate the subbasal nerve tracing assessed using the NeuronJ software (from images (**A**,**C**,**E**,**G**,**I**,**K**,**M**,**O**,**Q**,**S**) respectively). Red arrows (panels (**A**,**K**)) indicate a dendritic cell in the subbasal plexus. Note that dendritic cell density is higher in DED patients than in the control subjects.

IVCM image analysis revealed that patients with DED had a significantly higher corneal subbasal dendritic cell density compared with the control group (Figure 1, red arrows indicate dendritic cells). In addition, analysis of corneal nerve density with NeuronJ tracing revealed that patients with DED exhibited a lower corneal subbasal nerve density as compared to control subjects (16.5 ± 3.4 mm/mm^2^ for patients with DED vs. 20.3 ± 3.3 mm/mm^2^ for controls; *p <* 0.001). Images in Figure 1 show NeuronJ tracings overlaid on IVCM images of corneal nerve fibers (Figure 1). The IVCM results are summarized in Table 2.

### 2.3. Changes in Inflammatory Markers, Chemokines and Their Receptors, and Enkephalin mRNA Levels in Conjunctival Imprint Samples

**Figure 2:**
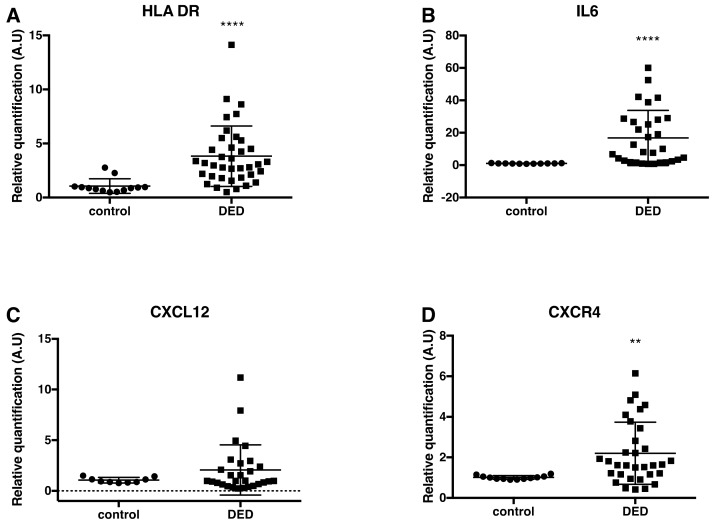

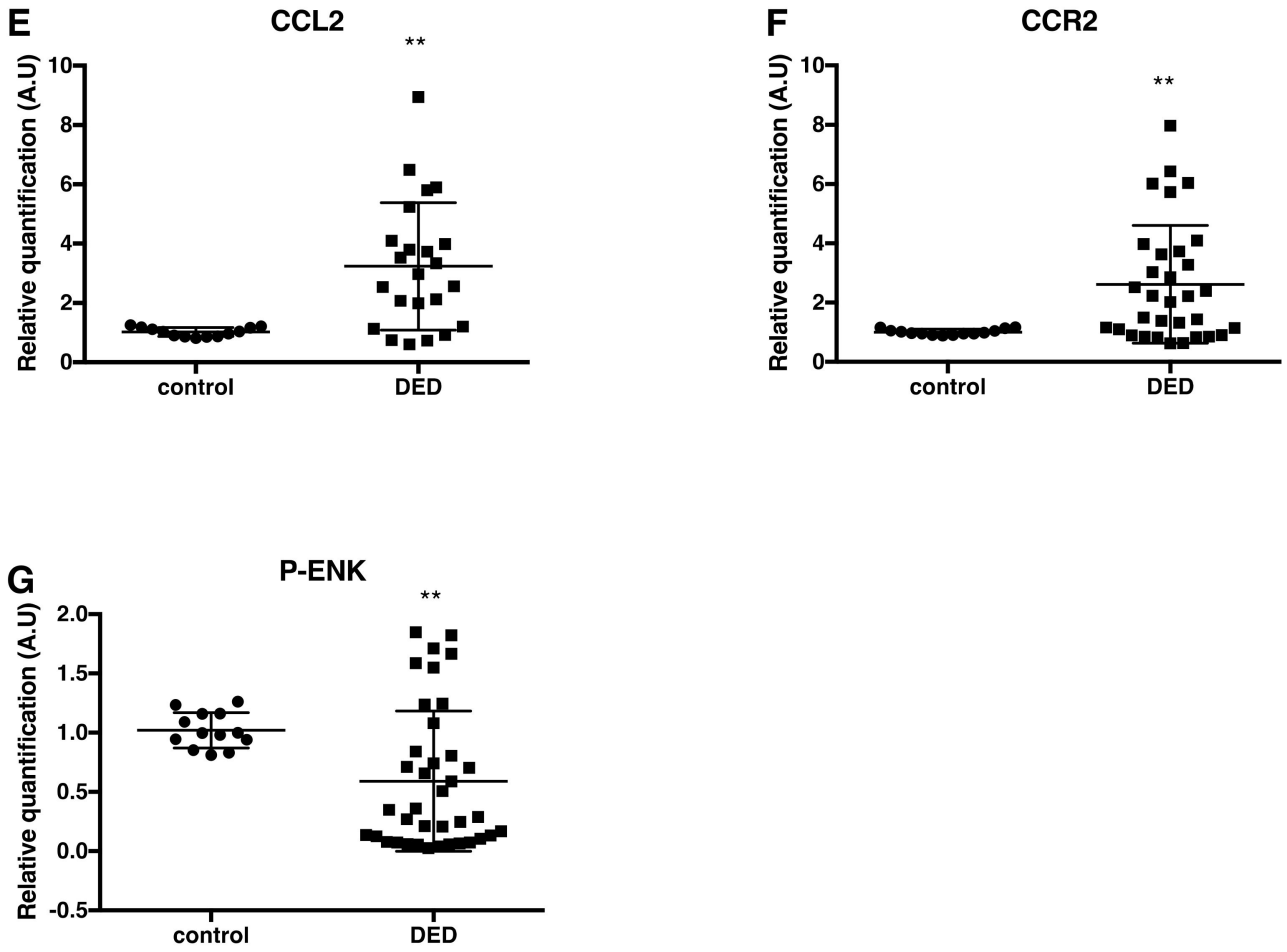
Scatter dot plots of relative quantification of *HLA-DR* (**A**) *IL-6* (**B**), *CXCL12* (**C**), *CXCR4* (**D**), *CCL2* (**E**), *CCR2* (**F**), and *P-ENK* (**G**) mRNA by RT-qPCR analysis in control subjects and patients with DED. The *HLA-DR* (*n =* 13 samples from controls; *n =* 37 samples from DED patients), *IL-6* (*n =* 11 samples from controls; *n =* 30 samples from DED patients), *CXCR4* (*n =* 12 samples from controls; *n =* 32 samples from DED patients), *CCL2* (*n =* 13 samples from controls; *n =* 23 samples from DED patients), and *CCR2* (*n =* 14 samples from controls; *n =* 32 samples from DED patients) mRNA levels of patients with DED were significantly higher than those in control subjects. In contrast, the *P-ENK* mRNA levels were significantly lower in DED patients (*n =* 38 samples) compared to those in control subjects (*n =* 13 samples). Statistical analyses between the two groups (controls and DED patients) were performed using the nonparametric Mann-Whitney test. ** *p <* 0.005; **** *p <* 0.0001 versus control. Data are expressed as mean ± standard deviation (SD). A.U = arbitrary unit.

We showed that *HLA-DR* and *IL-6* mRNA expression significantly increased by 3.82- and 16.75-fold, respectively, in DED patients as compared to control subjects (*p <* 0.0001); Figure 2A,B). mRNA expression of the chemokine *CCL2* and the chemokine receptors *CXCR4* and *CCR2* significantly increased by 3.23-, 2.20-, and 2.61-fold, respectively, in patients with DED as compared to control subjects (respectively, *p* = 0.0029, *p* = 0.001 and *p* = 0.007, respectively). In addition, a trend for a higher gene expression level of the chemokine *CXCL12* (the CXCR4 ligand) was found in subjects with DED compared to controls (Figure 2C–F), but this increase was not statistically significant. The *CXCL12* PCR primer used in this study did not specifically detect each *CXCL12* isoform, but recognized the main CXCL12 isoforms (alpha, beta, gamma, and delta).

Conversely, the gene expression levels of P-enkephalin (*P-ENK*, precursor of the endogenous opioid enkephalin) in the conjunctival impression cytology samples were significantly reduced by 40% in patients suffering from DED compared to control subjects (*p* = 0.003; Figure 2G).

### 2.4. Changes in Cell Morphology and in Inflammatory Markers in Conjunctiva-Derived Epithelial Cells Exposed to a Hyperosmolar Condition

**Figure 3:**
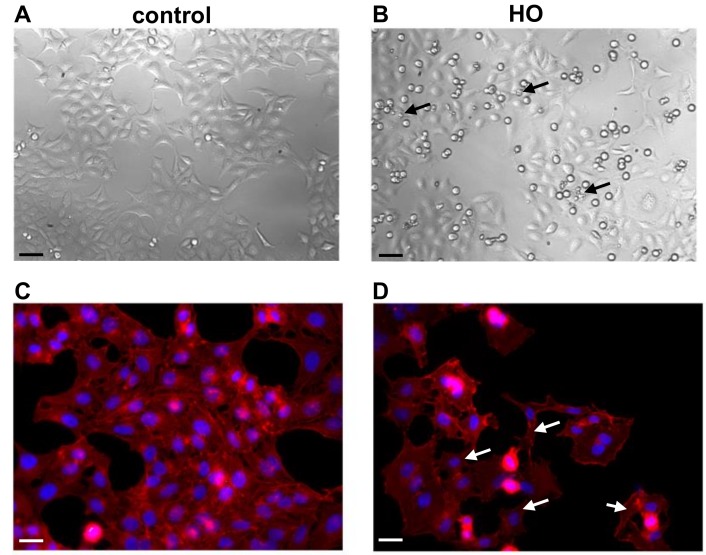
Phase contrast images and phalloidin staining of WKD cells cultured in Dulbecco’s Modified Eagle’s medium (DMEM) ((**A**,**C**) control condition) and in hyperosmolar (HO, 500 mOsm) condition. Note the morphological changes of WKD cells, the higher number of cell detachment with apoptotic bodies (black arrows), and the disruption of F-actin cytoskeleton (white arrows) under HO condition compared to control condition. Nuclear staining was assessed with DAPI (blue) and F-actin cytoskeleton staining with Alexa Fluor-546 phalloidin (red). Cells were stained after 24 h of incubation with medium (**A**,**C**), HO 500mOsm (**B**,**D**). Magnification ×100 (panels (**A**,**B**)) and ×400 (panels **C**,**D**). Black scale bars: 50 µm; white scale bars: 20 µm.

We next investigated whether a hyperosmolar condition (500 mOsm, HO500) could change the morphology of the conjunctiva-derived epithelial cells and induce a similar molecular signature to that observed in vivo on conjunctival imprints from DED patients. Phase contrast images of conjunctiva-derived epithelial cells showed that the hyperosmolar condition (HO500) leads to the appearance of cell detachment and apoptotic-like bodies (Figure 3B, black arrows) compared to the control condition (Figure 3A).

Detailed microscopic examination of the labeled cytoskeletal F-actin with rhodamine phalloidin revealed that the hyperosmolar condition induced changes in cell morphology and provoked the disruption of F-actin cytoskeleton (Figure 3D, white arrows) compared to the control condition (Figure 3C). Indeed, the F-actin cytoskeleton was severely disrupted, leading to cell detachment and death (Figure 3B, black arrows).

**Figure 4:**
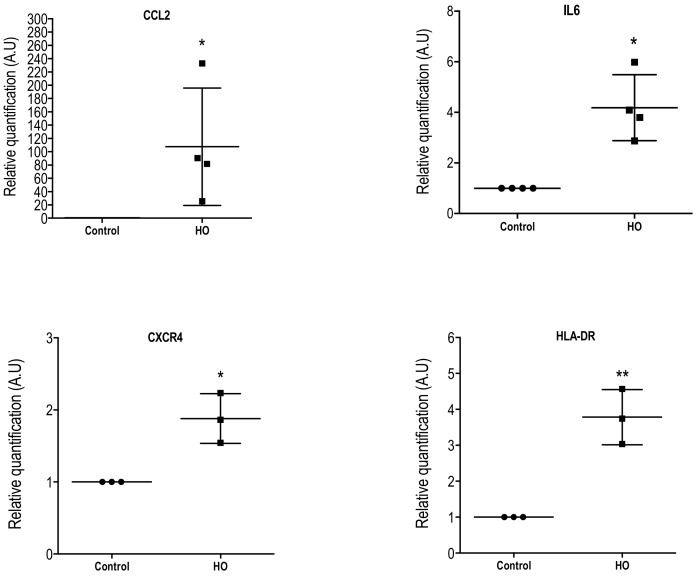
Scatter dot plots of relative quantification of *CCL2*, *IL-6*, *CXCR4*, and *HLA-DR* mRNA assessed using quantitative real-time RT-qPCR in WKD cells exposed to medium (control) or HO (500 mOsm) solutions for 24 h. Data were normalized using HPRT as a housekeeping gene. The *CCL2*, *CCL2*, *IL-6*, *CXCR4*, and *HLA-DR* mRNA levels were significantly higher than those measured in control condition. Data are expressed as mean ± standard deviation (SD) of a minimum of three independent experiments, determined in triplicate. Statistical analyses between the two groups (control and HO) were performed using the nonparametric Mann-Whitney test. * *p <* 0.05, ** *p <* 0.005 versus control condition. A.U = arbitrary unit.

Furthermore, the HO500 condition that was tested in this study significantly increased *CCL2* (X 107.6 ± 88.28; *p <* 0.05), *IL-6* (X 4.187 ± 1.30; *p <* 0.01), *CXCR4* (X 1.88 ± 0.34; *p <* 0.05), and *HLA-DR* (X 3.78 ± 0.76; *p <* 0.01) gene expression in conjunctiva-derived cells compared to control cells (Figure 4). In our experimental conditions, we were not able to detect *CXCL12*, *CCR2*, and *P-ENK* gene expression in both control and HO conditions, suggesting that *CCR2*, *CXCL12,* and *P-ENK* detected in the conjunctival imprints did not originate from conjunctival epithelial cells.

## 3. Discussion

In the present study, patients with DED and symptoms of ocular pain showed a significantly lower corneal nerve density and higher corneal dendritic cell density compared to normal subjects. Meanwhile, conjunctival impression cytology analysis revealed that *HLA-DR*, *IL-6*, *CXCR4*, and *CCL2/CCR2* mRNA levels were significantly increased in patients with DED compared to controls, whereas *P-ENK* mRNA levels were significantly decreased in the DED group. Similar results regarding inflammatory markers were obtained in vitro on immortalized human conjunctiva-derived epithelial cells challenged with an osmotic stress that mimics the dry eye condition. Altogether, these cellular and molecular analyses of the ocular surface suggested that proinflammatory molecules and endogenous enkephalin opioid have opposite gene regulation mechanisms during DED.

The innervation of the cornea and bulbar conjunctiva is mainly provided by the sensitive fibers originating from the ophthalmic region of the trigeminal ganglion [17]. Eye pain results from the stimulation of nociceptors expressed by nerve terminals of the trigeminal neurons, innervating the ocular surface. It has already been proposed that inflammation plays an important role in ocular surface pain [5,18]. Pro-inflammatory cytokines such as the interleukin IL-6 potentially contribute to increasing the activation of sensory nerve terminals, either by reducing their threshold for activation by sensory stimuli (sensitization) and/or by directly inducing or increasing their ongoing nerve activity [18,19]. Multicenter clinical trials have reliably demonstrated that the expression of *HLA-DR* significantly correlates with clinical signs and symptoms in DED [6]. Its expression by conjunctival cells can be considered as a promising biomarker of DED [6]. In the present study, RT-qPCR data showed a higher expression of *HLA-DR* and *IL-6* in conjunctival imprints from patients suffering from DED, confirming the central role of inflammation in this disease.

A previous elegant study from Barabino and colleagues detailed the immune response in the conjunctival epithelium from patients with dry eye disease [20]. Cytofluorimetry analysis revealed that the largest cell population includes conjunctival epithelial cells, while the smallest cell population includes CD45 leukocytes. The percentage of CD45+ cells in the whole cell population obtained in the dry eye versus the control group did not show significant differences. This result is in agreement with a previous study reported by the team [21]. The only leukocyte population that significantly increased (a twofold increase) in dry eye patients compared to controls was the monocyte/macrophage population (CD14+ cells). Whereas there was an absence of B cells (CD19−CD20−) in normal subjects and patients with DED, the percentage of NK cells (CD56+CD3−) was similar in control and patients suffering from DED [20]. In addition, the higher CD4/CD8 ratio and the increased percentages of CD14+ (monocytes/macrophages) observed in dry eye patients could be due to the effect of the conjunctival release of pro-inflammatory cytokines and chemokines.

We then focused the molecular analysis on two chemokine pairs, namely CXCL12/CXCR4 and CCL2/CCR2, known to play an important role in the modulation of inflammation and pain modulation [8,9,10]. In preclinical models of chronic pain (for example, models of inflammatory, neuropathic, or cancer-related pain), several studies have clearly demonstrated that these CXCL12 and CCL2 chemokines and their receptors are upregulated by neuronal and non-neuronal cells (astrocytes, microglial cells, and infiltrating immune cells) throughout nociceptive neuroanatomical pathways [13,22]. The molecular analysis of the conjunctival imprints revealed a significant increase of *CCL2* and its receptor *CCR2* in subjects with DED compared to control subjects. A previous experimental study proposed a potential treatment for DED using a CCR2 antagonist [23]. Topical administration of CCR2 antagonist reduced mRNA expression levels of interleukins, *IL-1α* and *IL-1β*, and *TNF-α* in the conjunctiva and cornea, thereby affecting the local proinflammatory environment in ocular surface tissues [23]. In addition, a study also reported the elevation of CCL2 in DED patient tears [7]. Altogether, these data and the present results strongly suggest activation of the CCL2–CCR2 axis in the ocular surface in dry eye patients. The chemokine receptor CCR2 is highly expressed by T lymphocytes and monocytes/macrophages, with the latter being doubled in dry eye patients [20]. This might explain the 2.61-fold increase of *CCR2* mRNA in conjunctival imprints from dry eye patients. Concerning the CCR2 ligand, the chemokine CCL2 is known to be expressed by epithelial cells, monocytes/macrophages, and lymphocytes [24]. These cellular expressions explain the 3.23-fold increase of *CCL2* mRNA observed in dry eye patients.

The CXCL12–CXCR4 axis has also been implicated in the modulation of somatic pain [8,25]. Molecular analysis of conjunctival imprints demonstrated a significant increase of *CXCR4* mRNA levels in patients suffering from DED. The present study only focuses on the CXCR4 receptor, in line with our previous work [11,22]. Therefore, the transcriptional level of the second CXCL12 receptor, CXCR7, recently named ACKR3, was not investigated. Concerning the CXCR4 ligand, we observed an increasing trend in mRNA *CXCL12* levels in dry eye patients. Here, we did not analyze the expression profile of the six *CXCL12* isoforms that have been identified in humans, but we investigated the expression of the main CXCL12 isoforms (alpha, beta, gamma, and delta).

Concerning the CXCL12/CXCR4 axis, it is well known that CD4+ T cells, epithelial cells, and human conjunctiva express high levels of CXCR4 [26]. Regarding CXCL12, this chemokine is widely distributed in many types of tissues and is constitutively expressed and secreted by monocytes and lymphocytes [27].

Again, these cell populations increased in dry eye patients, explaining the higher levels of CXCL12 and CXCR4 in patients suffering from DED.

In studies on DED, an association has been identified between tear film hyperosmolarity and inflammation severity elicited through receptor-induced increases in proinflammatory cytokine and chemokine release. Clinical studies have detected hyperosmolarity levels as high as 424 or 440 mOsm in severe DED patients [28,29]. A growing body of evidence implicates hyperosmotic stress as a potent inflammatory stimulus by triggering proinflammatory cytokine release and inflammation, as well as cell shrinkage. 

Here, we observed that a hyperosmolar condition (500 mOsm) induces cell detachment, F-actin disorganization. These results are in accordance with our previous study [30] reporting that HO500 induced a cell viability reduction of up to 80% at 24 h. Using the Hoechst 33342 assay, which provides a rapid and convenient assay for the apoptosis-based chromatin condensation that occurs during the late phase of apoptosis, we did not show any statistical difference between DMEM (control) and HO500 conditions, with only a slight nonsignificant increase in the HO500 condition. Moreover, compared to the control condition, caspase-3 activation was increased by only twofold in the HO500 condition, corresponding to an effective apoptotic process and explaining the slight increase in Hoechst staining [30]. However, despite these apoptotic patterns, WKD cells are still able to synthetize and secrete chemokines or cytokines such as *CCL2*, *CXCL8*, and *IL-6* [31]. Our previous study did not report significant differences in terms of cell viability or in DNA condensation (Hoechst assay) between the HO450 and HO500 conditions. Taking into account that previous studies on tear osmolarity in humans found a wide range of osmolarities in normal (290–330 mOsm/L) and dry eye patients (315–365 mOsm/L), even 675 mOsm/L, we found it pertinent to use the HO500 short-term exposure on WKD cells in line with our previous studies [30,31].

These data support the hypothesis that hypersomalority of the tear film could dramatically affect conjunctival cells and maintain or aggravate ocular surface impairment [30,31]. Furthermore, RTqPCR experiments showed that a hyperosmolar condition (500 mOsm) induced an increase of the chemokine *CCL2* and *IL-6* mRNA levels in vitro in conjunctiva-derived epithelial cells. The *CCL2* gene expression increase is in accordance with a previous study from our group [31]. Furthermore, we also found that *CXCR4* and *HLA-DR* gene expression was significantly increased (1.9- and 3.8-fold increase, respectively) compared to control cells for the HO condition.

Altogether, these in vitro data support the notion that in DED chemokines, proinflammatory cytokines and chemokine receptors drive a local inflammatory response that results in ocular tissue damage [5].

Chemokines and opioids are important regulators of immune, inflammatory, and neuronal responses in peripheral and central pain pathways. Several studies have demonstrated that there is an antagonistic action between proinflammatory chemokines and endogenous opioids in the modulation of somatic neuropathic pain [8,9,10,15]. Emerging studies describe how some chemokines such as CXCL12 and CCL2 are able to reduce acute opioid-mediated analgesia in the periaqueductal gray [32,33] and spinal cord [22,25], but to date there have been no data on trigeminal ocular pain.

In addition to its role in the central and peripheral nervous system, the precursor of the enkephalin gene (*P-ENK*) is expressed in immune cells that contribute to peripheral antinociception [12,34,35,36]. An additional role for these peptides emerged with the observation that opioid peptides released locally from immune cells can act on opioid receptors expressed on peripheral neurons to exert an antihyperalgesic effect in inflamed tissue [12,15,34]. In this study, we demonstrated that the detection of pro-enkephalin (*P-ENK*) mRNA, the main endogenous opioid, is feasible on conjunctival imprints. More interestingly, we report a significantly decreased expression of *P-ENK* in conjunctival cytology samples from patients suffering from DED. This result suggests that the local expression of the endogenous opioid system is probably downregulated during ocular pain. Furthermore, the fact that we did not detect *P-ENK* mRNA in the epithelial conjunctiva-derived WKD cell line, nor in the human corneal epithelial cell line (HCE) (data not shown), suggests that the source of the endogenous opioid does not come from epithelial cells but probably from immune cells. Further studies using primary human conjunctival epithelial cells could be used to confirm this result.

Knowing that enkephalin, the main endogenous opioid responsible for analgesia, is released by CD4(+) T lymphocytes [12], we cannot exclude the possibility that the downregulation of *P-ENK* mRNA levels observed in DED patients may result from an excess of enkephalin (negative regulatory loop) or the presence of CD4(+) T lymphocytes in deeper conjunctival epithelial layers. Another hypothesis would be that CD4(+) T cells could also be diluted by the presence of CD8(+) T lymphocytes, present in the majority of conjunctival epithelial samples.

This study has a number of limitations that should be pointed out. First, as the IVCM is a contact noninvasive examination, some patients agreed to a unilateral exam only, while other subjects agreed to undergo the exam bilaterally. Second, the quantity of the collected cells on conjunctival imprints determines the quantity of total extracted mRNA. For some samples, we obtained a low quantity of mRNA. This could result from the fact that conjunctival imprints requiring the contact of a filter paper on the conjunctiva are more difficult to perform in patients with ocular hypersensitivity. For these reasons, the number of samples or the number of patients were not always identical for IVCM examination and RTqPCR analysis. All the conjunctival imprints were used for transcriptional analysis, making this scientific achievement the first step to a better understanding of the pathophysiology of DED in relation to ocular surface pain. Unfortunately, this study does not allow us to evaluate the protein level of the different markers analyzed by RTqPCR, nor the immune composition. Further clinical studies are needed for these investigations and to evaluate the levels of these proteins in tears.

## 4. Material and Methods

### 4.1. Patients

This study was conducted at the Clinical Investigation Center (CIC 1423) of the Quinze-Vingts National Ophthalmology Hospital, Paris, France, with the approval of the Ile-de-France 5 Ethics Committee (#10793; approval date: 6 July 2010). All patients were informed of the aims of the study and their consent was obtained according to the Declaration of Helsinki. A total of 32 patients with symptomatic DED (23 women and nine men; mean age, 50.6 ± 3.4 years) and 15 control subjects (nine women and six men; mean age, 50.7 ± 7.2 years) were recruited.

DED was defined as a Schirmer 1 test <5 mm and/or tear film break-up time (TBUT) <10 s, accompanied by complaints of ocular irritation in the absence of other ocular or systemic diseases (diabetes, thyroid disease, arthritis, etc.) [37]. The Ocular Surface Disease Index (OSDI) questionnaire was used to grade subjective dry eye symptoms [38]. The overall OSDI score defined the ocular surface as normal (0–12 points) or as having mild (13–22 points), moderate (23–32 points), or severe (33–100 points) disease.

A visual analog scale was used to quantify the pain associated with DED (the score ranges from 0 to 10, with 0 being the absence of pain and 10 the maximum possible pain). All patients with DED had a score ≥1/10. Then, all patients underwent a complete examination of the ocular surface of in the following order: TBUT, corneal, and conjunctival fluorescein staining to provide an Oxford scale ranging from 0 (no keratitis) to 5 (severe keratitis) [39], and a Schirmer test. Then, in vivo confocal microscopy (IVCM) analysis of the central corneal subbasal nerves and dendritic cell density was performed. 

Finally, we collected conjunctival cells using conjunctival impression cytology as previously described [6].

All control subjects had no complaint of ocular surface irritation and no anterior segment abnormality on biomicroscopic examination and ocular surface tests. The exclusion criteria for this study were: age under 18 years, subjects unable to complete the questionnaire or understand the procedures, the presence of ocular or systemic disease or the use of topical or systemic medications that may affect the cornea and the ocular surface (except the use of unpreserved tear substitutes in the DED group), previous eye surgery, or contact lens wear.

### 4.2. In Vivo Confocal Microscopy

IVCM of the cornea was performed using the Rostock Cornea Module^®^ of the Heidelberg Retina Tomograph^®^ (HRT/RCM) (Heidelberg Engineering GmbH, Heidelberg, Germany). This noninvasive imaging technique analyzes the ocular surface structures and in particular corneal nerves, with a histology-like resolution [40]. The images comprised 384 × 384 pixels covering an area measuring 400 × 400 µm with a 2-µm transverse optical resolution, a 4-µm axial optical resolution, and a 0.024-s acquisition time (Heidelberg Engineering). Central cornea IVCM images (approximately 50) were acquired by focusing from the superficial epithelium to the anterior stroma and using the same illumination intensity (manual mode). Firstly, we studied the density of dendritic cells located beneath the epithelium of each patient, in the anterior stroma. Dendritic cells corresponded to hyper-reflective dendritiform structures differentiated from corneal subbasal nerves. Secondly, as described previously [40], the density of subbasal nerves was evaluated (expressed in mm/mm^2^). All parameters were analyzed and calculated retrospectively using Image J with NeuronJ^®^ software by a single researcher (P.N.) who was blinded to patient identity and the results of the ocular surface investigations. NeuronJ is a free ImageJ plug in (National Institutes of Health, Bethesda, MD, USA) to facilitate the tracing and quantification of elongated image structures such as nerves in 2D images. The density of subbasal nerves was defined as the total length of the nerves visible within a frame. For the quantification of the dendritic cells, the number of positive cells was counted manually by an experimenter who was blind to the experimental design. For each eye whenever possible and for each parameter analyzed, the result was the average of the analysis of five randomly selected images. Data from the right and left eyes were then pooled to provide a mean value.

### 4.3. Conjunctival Impression Cytology

Conjunctival cells were collected using impression cytology as previously described [6]. This method consists of gently applying a polyethersulfone filter (Supor^®^, Gelman, Pall Science, Ann Arbor, MI, USA) to the superior conjunctiva to collect the superficial conjunctival cells. This minimally invasive sample was taken bilaterally whenever possible for each patient after instillation of a drop of topical anesthetic (oxybuprocaine, 1.6 mg/0.4 mL, Thea Laboratory, Clermont-Ferrand, France). Conjunctival imprints were placed in a sterile tube and stored at −80°C until analysis.

### 4.4. WKD Cell Lines

The Wong Kilbourne derivative of the Chang (WKD) HeLa-modified conjunctiva-derived epithelial cell line (clone 1-5c-4, American Type Culture Collection (ATCC)-certified cell line (CCL), 20.2) was cultured under classic conditions (moist atmosphere, 5% CO_2_, 37 °C) in Dulbecco minimum essential medium (DMEM) supplemented with 10% fetal bovine serum, 1% glutamine (200 mM), 1% penicillin (10,000 units/mL), and streptomycin (10,000 μg/mL) for 24 h to reach 80% confluence. Confluent cells were suspended in cell culture medium after trypsin incubation and seeded in six-well culture dishes and slides at a density of 100,000 cells per well and kept at 37 °C for 24 h. All reagents for cellular culture were purchased from Gibco (Gibco, Life Technologies, Carlsbad, CA, USA). A study comparing the gene expression profiles of conjunctival cell lines, including WKD cells, with primary cultured conjunctival epithelial cells and human conjunctival tissue, explained that these cell lines may be used to investigate specific cellular mechanisms such as those related to inflammation [41].

### 4.5. Hyperosmolar Condition and Cell Stimulation Protocol

At confluence, WKDs were grown for 24 h, in: (1) supplemented DMEM considered as the control condition (340 mosm), (2) hyperosmolar medium achieved by adding NaCl (Sigma-Aldrich, Saint-Quentin-Fallavier, France) in supplemented DMEM (500 mOsm), called HO (HO500), as previously described [25,26]. The osmolarity value was assessed using an osmometer (Roebling 13DR, Berlin, Germany). Cell exposure to hyperosmolar conditions of 500 mOsm for 1 h are classic protocols used in in vitro studies on DED [41,42]. After this incubation period, the culture medium was changed and cells were cultured for 24 h. For Phalloidin staining, WKDs were grown on round sterile cover glasses (diameter, 14 mm; Menzel GmbH, Germany), challenged or not challenged with hyperosmolar medium (500 mOsm) for 1 h. After this incubation period, the culture medium was changed and cells were cultured for 24 h and fixed in 4% paraformaldehyde. Cells were then incubated with phalloidin (1:40, Alexa Fluor^®^ 546) and DAPI (1:2000) for 1 h and rinsed in PBS. Cover glasses were then mounted on glass microscope slides before observation with a Zeiss M1 epifluorescence microscope.

### 4.6. RT-qPCR Analysis

mRNAs were extracted from conjunctival imprints and the WKD cell line using the NucleoSpin^®^ RNA XS extraction kit (Macherey-Nagel, Hoerdt, Germany). The total RNA elution volume was 11 µL. The quality and the concentration of mRNAs were then measured using a NanoDrop (Thermo Scientific, Labtech, Uckfield, UK). Reverse transcription was performed with 660 ng of RNA using High-Capacity cDNA Reverse Transcription (Applied Biosystems, Foster City, CA, USA) according to the manufacturer’s instructions. The cDNA was then diluted in DNase/RNase-free water in a final concentration of 5 ng·μL^−1^. The quantitative real-time PCR was performed with 25 ng of cDNA added to 15 µL of PCR Master Mix (Applied Biosystems Master Mix; TaqMan Universal PCR Master Mix, Applied Biosystems) in a final volume of 20 µL. The real-time PCR was amplified in duplicate for each sample and followed in real time using the 7300 Real-Time PCR System (Applied Biosystems), with ABgene Absolute QPCR ROX Mix (Thermo Fisher Scientific, Waltham, MA, USA). The probes used were: *HLA-DR α* (Hs00219575_m1), *P-ENK* (Hs00175049_m1), *CXCL12* (Hs00171022_m1), *CXCR4* (Hs00237052_m1), *CCR2* (Hs00356601_m1), *CCL2* (Hs00234140_m1), *IL-6* (Hs00985639_m1), and glyceraldehyde-3-phosphate dehydrogenase (*GAPDH*) (Hs99999905_m1) or *HPRT* (Hs02800695_m1) as housekeeping genes. The specific cDNA level was calculated after normalization of the result for each sample relative to the expression level of the *GAPDH* or *HPRT* reporter genes. The data are presented as relative mRNA units with respect to control values.

### 4.7. Statistics

Statistical analyses between two groups were performed using the nonparametric Mann-Whitney test using GraphPad Prism 6.0 for Mac OS X (GraphPad Software, La Jolla, CA, USA, www.graphpad). Data are expressed as mean ± Standard Deviation (SD). The number of experiments is specified in the figure legends. *p*-Values shown as * *p <* 0.05, ** *p <* 0.005, and **** *p <* 0.0001 were considered statistically significant.

## 5. Conclusions

Altogether, these molecular analyses of the ocular surface from patients with dry eye pain demonstrate that proinflammatory molecules and the endogenous enkephalin opioid have opposite gene regulation during dry eye pain. A better understanding of the sequence and nature of the events that drive these molecular mechanisms will offer significant promise for the discovery of new mechanisms and targets for the management of chronic ocular pain during dry eye.

## Figures and Tables

**Table 1:** Demographic and clinical test results.

Parameters	Control	DED	*p*-Values
Age (years)	50.7 ± 24.0	50.6 ± 19.6	0.98
Sex, female/male (%)	60/40	72/28	
Symptoms (years)	0 ± 0	5.0 ± 4.7	<0.01
OSDI score	6.2 ± 4.4	67.8 ± 21.4	<0.01
Schirmer test (mm)	24.7 ± 4.1	12.5 ± 8.8	<0.02
Break-up time (s)	14.4 ± 2.3	5.4 ± 3.3	<0.01
Oxford score	0 ± 0	1.4 ± 1.6	<0.01
Assessment of pain (/10)	0 ± 0	6.1 ± 2.9	<0.01

Fifteen control subjects and 32 patients with DED were enrolled in this clinical study. Data are expressed as mean ± standard deviation (SD). Statistical analyses between the two groups were performed using the nonparametric Mann-Whitney test. Level of statistical significance, *p* < 0.05. OSDI: Ocular Surface Disease Index.

**Table 2:** IVCM analysis of corneal subbasal dendritic cells and corneal subbasal nerve density.

IVCM Parameters	Control	DED	*p*-Values
Subbasal dendritic cell density (c/mm^2^)	49.2 ± 43.7	103.5 ± 84.5	<0.01
Nerve density (mm/mm^2^)	20.3 ± 3.3	16.5 ± 3.4	<0.01

Subbasal dendritic cell density and nerve density were analyzed in IVCM images from 15 control subjects and 25 patients with DED. The data represent the average from the right and left eyes. Patients with DED exhibit a higher dendritic cell density and lower corneal subbasal nerve density than control subjects. Data are expressed as mean ± standard deviation (SD). Statistical analyses between the two groups were performed using the nonparametric Mann-Whitney test. Level of statistical significance, *p* < 0.05. IVCM: *in vivo* confocal microscopy; c: cells.

## References

[1] Deschamps N., Ricaud X., Rabut G., Labbe A., Baudouin C., Denoyer A. (2013). The impact of dry eye disease on visual performance while driving. Am. J. Ophthalmol..

[2] Labbe A., Wang Y.X., Jie Y., Baudouin C., Jonas J.B., Xu L. (2013). Dry eye disease, dry eye symptoms and depression: The beijing eye study. Br. J. Ophthalmol..

[3] Schiffman R.M., Walt J.G., Jacobsen G., Doyle J.J., Lebovics G., Sumner W. (2003). Utility assessment among patients with dry eye disease. Ophthalmology.

[4] Sullivan B.D., Crews L.A., Messmer E.M., Foulks G.N., Nichols K.K., Baenninger P., Geerling G., Figueiredo F., Lemp M.A. (2014). Correlations between commonly used objective signs and symptoms for the diagnosis of dry eye disease: Clinical implications. Acta Ophthalmol..

[5] Bron A.J., de Paiva C.S., Chauhan S.K., Bonini S., Gabison E.E., Jain S., Knop E., Markoulli M., Ogawa Y., Perez V. (2017). Tfos dews ii pathophysiology report. Ocul. Surf..

[6] Brignole-Baudouin F., Riancho L., Ismail D., Deniaud M., Amrane M., Baudouin C. (2017). Correlation between the inflammatory marker HLA-DR and signs and symptoms in moderate to severe dry eye disease. Investig. Ophthalmol. Vis. Sci..

[7] Na K.S., Mok J.W., Kim J.Y., Rho C.R., Joo C.K. (2012). Correlations between tear cytokines, chemokines, and soluble receptors and clinical severity of dry eye disease. Investig. Ophthalmol. Vis. Sci..

[8] Reaux-Le Goazigo A., Rivat C., Kitabgi P., Pohl M., Melik Parsadaniantz S. (2012). Cellular and subcellular localization of cxcl12 and cxcr4 in rat nociceptive structures: Physiological relevance. Eur. J. Neurosci..

[9] Van Steenwinckel J., Auvynet C., Sapienza A., Reaux-Le Goazigo A., Combadiere C., Melik Parsadaniantz S. (2015). Stromal cell-derived CCL2 drives neuropathic pain states through myeloid cell infiltration in injured nerve. Brain Behav. Immun..

[10] Van Steenwinckel J., Reaux-Le Goazigo A., Pommier B., Mauborgne A., Dansereau M.A., Kitabgi P., Sarret P., Pohl M., Melik Parsadaniantz S. (2011). CCL2 released from neuronal synaptic vesicles in the spinal cord is a major mediator of local inflammation and pain after peripheral nerve injury. J. Neurosci..

[11] Melik Parsadaniantz S., Rivat C., Rostene W., Reaux-Le Goazigo A. (2015). Opioid and chemokine receptor crosstalk: A promising target for pain therapy?. Nat. Rev. Neurosci..

[12] Basso L., Boue J., Mahiddine K., Blanpied C., Robiou-du-Pont S., Vergnolle N., Deraison C., Dietrich G. (2016). Endogenous analgesia mediated by cd4(+) t lymphocytes is dependent on enkephalins in mice. J. Neuroinflamm..

[13] Roques B.P., Fournie-Zaluski M.C., Wurm M. (2012). Inhibiting the breakdown of endogenous opioids and cannabinoids to alleviate pain. Nature reviews. Drug Discov..

[14] Stein C., Kuchler S. (2013). Targeting inflammation and wound healing by opioids. Trends Pharmacol. Sci..

[15] Poras H., Bonnard E., Dange E., Fournie-Zaluski M.C., Roques B.P. (2014). New orally active dual enkephalinase inhibitors (DENKIs) for central and peripheral pain treatment. J. Med. Chem..

[16] Stein C., Kuchler S. (2012). Non-analgesic effects of opioids: Peripheral opioid effects on inflammation and wound healing. Curr. Pharm. Des..

[17] Marfurt C.F., Cox J., Deek S., Dvorscak L. (2010). Anatomy of the human corneal innervation. Exp. Eye Res..

[18] Belmonte C., Nichols J.J., Cox S.M., Brock J.A., Begley C.G., Bereiter D.A., Dartt D.A., Galor A., Hamrah P., Ivanusic J.J. (2017). Tfos dews ii pain and sensation report. Ocul. Surf..

[19] Acosta M.C., Luna C., Quirce S., Belmonte C., Gallar J. (2013). Changes in sensory activity of ocular surface sensory nerves during allergic keratoconjunctivitis. PAIN.

[20] Barabino S., Montaldo E., Solignani F., Valente C., Mingari M.C., Rolando M. (2010). Immune response in the conjunctival epithelium of patients with dry eye. Exp. Eye Res..

[21] Baudouin C., Liang H., Bremond-Gignac D., Hamard P., Hreiche R., Creuzot-Garcher C., Warnet J.M., Brignole-Baudouin F. (2005). CCR 4 and CCR 5 expression in conjunctival specimens as differential markers of T(H)1/T(H)2 in ocular surface disorders. J. Allergy Clin. Immunol..

[22] Rivat C., Sebaihi S., van Steenwinckel J., Fouquet S., Kitabgi P., Pohl M., Melik Parsadaniantz S., Reaux-Le Goazigo A. (2014). Src family kinases involved in CXCL12-induced loss of acute morphine analgesia. Brain Behav. Immun..

[23] Goyal S., Chauhan S.K., Zhang Q., Dana R. (2009). Amelioration of murine dry eye disease by topical antagonist to chemokine receptor 2. Arch. Ophthalmol..

[24] Berman J.W., Guida M.P., Warren J., Amat J., Brosnan C.F. (1996). Localization of monocyte chemoattractant peptide-1 expression in the central nervous system in experimental autoimmune encephalomyelitis and trauma in the rat. J. Immunol..

[25] Wilson N.M., Jung H., Ripsch M.S., Miller R.J., White F.A. (2011). Cxcr4 signaling mediates morphine-induced tactile hyperalgesia. Brain Behav. Immun..

[26] Dieckow J., Brandt W., Hattermann K., Schob S., Schulze U., Mentlein R., Ackermann P., Sel S., Paulsen F.P. (2016). CXCR4 and CXCR7 Mediate TFF3-Induced Cell Migration Independently From the ERK1/2 Signaling Pathway. Investig. Ophthalmol. Vis. Sci..

[27] Sánchez-Martín L., Estecha A., Samaniego R., Sánchez-Ramón S., Vega M.Á., Sánchez-Mateos P. (2011). The chemokine CXCL12 regulates monocyte-macrophage differentiation and RUNX3 expression. Blood.

[28] Farris R.L. (1994). Tear osmolarity—A new gold standard?. Adv. Exp. Med. Biol..

[29] Gilbard J.P., Farris R.L., Santamaria J. (1978). Osmolarity of tear microvolumes in keratoconjunctivitis sicca. Arch. Ophthalmol..

[30] Clouzeau C., Godefroy D., Riancho L., Rostene W., Baudouin C., Brignole-Baudouin F. (2012). Hyperosmolarity potentiates toxic effects of benzalkonium chloride on conjunctival epithelial cells in vitro. Mol. Vis..

[31] Warcoin E., Clouzeau C., Roubeix C., Raveu A.L., Godefroy D., Riancho L., Baudouin C., Brignole-Baudouin F. (2017). Hyperosmolarity and benzalkonium chloride differently stimulate inflammatory markers in conjunctiva-derived epithelial cells in vitro. Ophthalmic Res..

[32] Szabo I., Chen X.H., Xin L., Adler M.W., Howard O.M., Oppenheim J.J., Rogers T.J. (2002). Heterologous desensitization of opioid receptors by chemokines inhibits chemotaxis and enhances the perception of pain. Proc. Natl. Acad. Sci. USA.

[33] Chen X., Geller E.B., Rogers T.J., Adler M.W. (2007). Rapid heterologous desensitization of antinociceptive activity between mu or delta opioid receptors and chemokine receptors in rats. Drug Alcohol Depend..

[34] Cabot P.J., Carter L., Schafer M., Stein C. (2001). Methionine-enkephalin-and dynorphin a-release from immune cells and control of inflammatory pain. PAIN.

[35] Schafer M., Carter L., Stein C. (1994). Interleukin 1 beta and corticotropin-releasing factor inhibit pain by releasing opioids from immune cells in inflamed tissue. Proc. Natl. Acad. Sci. USA.

[36] Mousa S.A., Shakibaei M., Sitte N., Schafer M., Stein C. (2004). Subcellular pathways of beta-endorphin synthesis, processing, and release from immunocytes in inflammatory pain. Endocrinology.

[37] Stapleton F., Alves M., Bunya V.Y., Jalbert I., Lekhanont K., Malet F., Na K.S., Schaumberg D., Uchino M., Vehof J. (2017). TFOS DEWS II epidemiology report. Ocul. Surf..

[38] Schiffman R.M., Christianson M.D., Jacobsen G., Hirsch J.D., Reis B.L. (2000). Reliability and validity of the Ocular Surface Disease Index. Arch. Ophthalmol..

[39] Bron A.J., Evans V.E., Smith J.A. (2003). Grading of corneal and conjunctival staining in the context of other dry eye tests. Cornea.

[40] Labbe A., Liang Q., Wang Z., Zhang Y., Xu L., Baudouin C., Sun X. (2013). Corneal nerve structure and function in patients with non-sjogren dry eye: Clinical correlations. Investig. Ophthalmol. Vis. Sci..

[41] Tong L., Diebold Y., Calonge M., Gao J., Stern M.E., Beuerman R.W. (2009). Comparison of gene expression profiles of conjunctival cell lines with primary cultured conjunctival epithelial cells and human conjunctival tissue. Gene Expr..

[42] Zheng Q., Ren Y., Reinach P.S., Xiao B., Lu H., Zhu Y., Qu J., Chen W. (2015). Reactive oxygen species activated NLRP3 inflammasomes initiate inflammation in hyperosmolarity stressed human corneal epithelial cells and environment-induced dry eye patients. Exp. Eye Res..

